# Improving the Tolerance to Salinity Stress in Lettuce Plants (*Lactuca sativa* L.) Using Exogenous Application of Salicylic Acid, Yeast, and Zeolite

**DOI:** 10.3390/life12101538

**Published:** 2022-10-03

**Authors:** Mahfoud Babaousmail, Mohammed S. Nili, Rania Brik, Mohammed Saadouni, Sawsan K. M. Yousif, Rihab M. Omer, Nahid A. Osman, Abdulaziz A. Alsahli, Hatem Ashour, Ahmed M. El-Taher

**Affiliations:** 1 Laboratory of Biodiversity and Biotechnology Applications in Agriculture, University of El Oued, El Oued 39000, Algeria; 2 Department of Agricultural Sciences, University of El Oued, El Oued 39000, Algeria; 3 Department of Chemistry, College of Arts and Science in Baljurashi, Al-Baha University, Al Bahah 65528, Saudi Arabia; 4 Department of Science and Technology, Ranya Collage, Taif University, Taif 21944, Saudi Arabia; 5 Department of Botany & Microbiology, Science College, King Saud University, Riyadh 11451, Saudi Arabia; 6 Department of Agricultural Botany, Faculty of Agriculture, Ain Shams University, Cairo 11566, Egypt; 7 Department of Agricultural Botany, Faculty of Agriculture, Al-Azhar University, Cairo 71524, Egypt

**Keywords:** abiotic stress, aluminosilicate, biostimilants, osmoprotectants, plant resistance, *Saccharomyces cerevisiae*

## Abstract

Salinity is among the most limiting factors of crop production worldwide. This study aims to investigate the influence of the exogenous application of zeolite, yeast, and salicylic acid in alleviating the negative effect of salt stress under field conditions. Lettuce plants (*Lactuca sativa* L. cv. Batavia) were tested in a split-plot arrangement replicated three times. The salt stress was applied as a whole-plot factor in the concentrations (0 mM, 50 mM, 100 mM, and 150 mM NaCl). After 28 days of sowing, the plants were sprayed twice during the foliage growth with (control, salicylic acid 0.02%, yeast extract 3%, and zeolite 0.5%) as a split-plot factor. The length of roots and shoots, the number and area of leaves, and the biomass accumulation (dry and fresh weights) were measured 50 days after sowing. The concentrations of total soluble sugars, proline, Chlorophylls a and b in leaves have also been quantified. Salt stress significantly reduced the growth and the total chlorophyll of the lettuce plants (*p* < 0.05) and increased their proline and sugar contents’. Zeolite application improved the growth of lettuce at 0 and 50 mM NaCl, but at the highest salinity level only the number of leaves was improved by 15%. At a mild salinity stress, the application of salicylic acid has significantly (*p* < 0.05) increased the root length, height of plant, chlorophyll, and proline contents. Regarding the high stress levels (100 and 150 mM NaCl), yeast application showed the best tolerance to salinity stress by improving significantly most of the growth parameters (*p* < 0.05) but with lower proline, sugar, and chlorophyll contents. In general, foliar spray of yeast extract may offer a good alternative source of nutrients through leaves, leading to a better tolerance of the high salt stress exerted on roots.

## 1. Introduction

Among the abiotic stresses, salinity is the most harmful factor that seriously reduces plant productivity. Salinity is a significant threat to modern agriculture because it inhibits and impairs the growth and development of plants [1]. It is indeed predicted that saline water covers at least 70% of the Earth’s surface [2,3]. However, due to the annual increase of soil salt in the root zone [4], salinity can severely limit crop production, particularly in arid and semi-arid lands [5].

Plants growing under saline conditions have been reported to be affected in three ways: reduced water potential in the rhizosphere causing water shortage, phytotoxicity of ions such as Na^+^ and Cl^−^, and imbalance of nutrients reducing their absorption and transport in plants [6]. Consequently, salinity influences all of the main processes, such as growth, photosynthesis, and metabolism reactions [7].

Plants have developed different mechanisms to resist salt stress [8]. These mechanisms may be grouped into three categories: net exclusion of toxic ions from the shoot; tissue tolerance, by compartmentalization of Na+ ions in parts of the plant to avoid excessive accumulation of toxic ions near metabolic reactions; and shoot ion-independent tolerance, the mechanism that focuses on maintaining growth and water absorption regardless of the level of sodium accumulation in the shoot [9]. Besides endogenous mitigating strategies, exogenous strategies could use chemical, physical, and biological applications in plants or seeds [10]. For example, an increasing number of studies have demonstrated the beneficial effect of the exogenous application of salicylic acid, yeast, or zeolite in alleviating the adverse effect of salt stress. 

Natural zeolites (ZEO; clay minerals of the aluminosilicate group) are used as inorganic soil conditioners and are commonly used in agriculture as a silicon source to improve the growth and yield of crops productivity under normal or stressful conditions [11,12]. Zeolite alleviated salt toxicity symptoms in strawberries and resulted in increased yield due to the better hydric status of the plant [13]. Zeolite amendment mitigated salt stress and improved plant growth of Kentucky bluegrass *Poa pratensis* [14]. Beneficial effects of zeolite were also concluded by Aboul-Magd et al. (2020) [15] on maize plants grown under saline conditions where zeolite improved turf quality and the uptake of nutrients by the plants. 

Salicylic acid (SA) is a joint plant-produced organic compound and a potential endogenous plant hormone that plays a vital role in the growth and development of plants [16]. The role of SA and its involvement in plant resistance to salinity is widely studied. The salicylic acid application reduced the inhibitory effect of salt stress on cowpea plants (*Vigna unguiculata*), by enhancing the growth, physiological and anatomical properties. [17] and improved the performance of wheat seedlings under salt stress [18]. In addition, SA application stimulates salt resistance in maize plants by improving the photosynthesis rate and the metabolism of carbohydrates [19], it also induces energy production and antioxidant enzymes in chickpeas [20]. 

Yeast (YST) *Saccharomyces cerevisiae* is a biofertilizer; due to its advantageous effects on plants and its safety for human health and the environment, yeast could be used in soil or foliar application on many crops [21,22]. It is considered a cost-effective biofertilizer that enhances plant nutrition and vigor and helps resist abiotic stress [23]. Yeast has been proven to be effective in alleviating salt stress effects on cucumber [24], broad bean (*Vicia faba* L.) [25], tomato [26], and date palm [27]. The enhancements of plant development when the active dry yeast is applied can be attributed to its different nutrient contents, such as minerals, amino acids, vitamins, and phytohormones, especially cytokinins [22,28]. The availability of these nutrients which are usually involved in resistance mechanisms helps plants to grow better under saline conditions [29,30]. 

Lettuce (*Lactuca sativa* L.) is a fresh vegetable commonly used in salad mixtures and sandwiches. It plays an essential role due to its nutritional value on proteins, vitamins, and cellulose [31,32,33,34]. The top 100 lettuce-producing countries in the world include 14 African countries [35]. In sub-Saharan Africa, pteridophytes, such as lettuce, can be used to help provide food security during drought periods [36]. 

It would be of particular interest to investigate the effect of using salicylic acid, yeast, and zeolite and compare their impacts on lettuce plants under different salinity levels. We hypothesize that the application of these preparations will enhance plant performance. However, the plant response will vary in many growth and biochemical aspects. To verify this hypothesis, this study was carried out to measure lettuce plants’ growth and biochemical attributes.

## 2. Materials and Methods

### 2.1. Study Site

The experiment was conducted under field conditions on a private farm in In Zghmir (Lat: 27,094562 Long: −0,1252864), Adrar, Algeria. The Adrar region has a hot arid climate. The annual rainfall recorded in 2021 was 8.87 mm, with an average daily temperature of 27.3 °C. (https://en.tutiempo.net/climate/2021/ws-606200.html accessed on 20 January 2022). 

### 2.2. Experimental Design and Plant Growth 

The study followed a randomized complete block design with a split-plot arrangement that replicated nine plants three times per biological replication. The seeds of lettuce *Lactuca Sativa* L. (Blonde of Paris Batavia) cultivar were sown directly in the field on 16 March 2021. After 25 days of sowing, the salt stress was applied by a daily irrigation of 2 liters per plant for two weeks as a whole-plot factor in the following concentrations (0 mM, 50 mM, 100 mM, and 150 mM NaCl). On day 26 after sowing, the lettuce plants were sprayed with treatments (control, salicylic acid, yeast, and zeolite) as a split-plot factor. A second treatment spraying was done again with an interval of two weeks. The sprays were done in the early mornings with an approximate volume of 15 ml per plant.

### 2.3. Treatments Preparation

- Control: Distilled water.
- Salicylic acid: 0.2 g L^−1^ prepared by dissolving 0.2 grams of salicylic acid, obtained from Sigma-Aldrich Chemical Company in 1 liter of distilled water.
- Yeast: A 3% yeast extract was prepared using a commercial baker’s yeast (*Saccharomyces cerevisiae*) obtained from Saf-instant® France by dissolving a quantity of dry yeast in distilled water, a 1:1 ratio of sugar was added (as a source of C and N). The culture was grown for 48 h at 28 °C before application to the plants [26].
- Zeolite: To prepare a concentration of 5 g L^−1^, a quantity of 5 grams of natural zeolite mineral obtained from Rota Mining Corporation was dissolved in 1 liter of distilled water.

### 2.4. Growth Attributes

The plants were harvested after 50 days of sowing. The length of roots and shoots was measured and the number of leaves was visually counted. The fresh weights of roots and leaves were recorded using a balance. Dry mass of leaves and roots was determined after drying at 60 °C. Estimates of leaf area were obtained by the equation, leaf area (cm^2^) = x/y, where x is the weight (g) of the area covered by the leaf outline on a millimeter graph paper, and y is the weight of one cm^2^ of the same graph paper.

### 2.5. Estimation of Photosynthetic Pigments

Leaf photosynthetic pigments were assessed using an acetone method [37]. Leaves (0.5 g) were ground, and the powder was extracted with 80% acetone for 30 min in the dark. Plant extracts were centrifuged at 10,000 × g for 20 min. The amounts of chlorophyll a and b were measured in a spectrophotometer (Shimadzu UV–vis 1800) at 645 and 663 nm using the following formula:

Chl a (mg^−1^ FW) = (12.7 × A663 − 2.69 × A645) × V/(1000 × W)

Chl b (mg g^−1^ FW) = (22.9 × A645 − 4.68 × A663) × V/(1000 × W)

where V is the volume of 80% (*v*/*v*) acetone (mL), and W is the fresh weight (FW) of sample (g).

*Total chlorophyll content* (mg^−1^ FW) = Chl a + Chl b.

### 2.6. Estimation of Total Leaf Soluble Sugar

Total carbohydrates were determined based on the method of phenol sulfuric acid as described by DuBois et al. [38]. Pure glucose was used as standard, and the number of total carbohydrates was expressed as µg glucose 100g^−1^ fresh weight. The absorbance is read in a Shimadzu UV–vis 1800 spectrophotometer at a wavelength of 490 nm. The total sugar was calculated following the formula:

Sugar (mg 100 mg^−1^) = Absorbance of sample/Absorbance Standard × [Standard] × dilution factor.

### 2.7. Estimation of Total Leaf Proline

Proline was quantified using the technique of Troll [39]. Fresh leaf samples (0.5 g) were chopped and homogenized with 1× volume of 100 mM sodium phosphate buffer (pH 6.0). The sample was centrifuged at 16,000× *g* for 10 min. The reaction mixture contained 200 μl supernatant and 1 mL ninhydrin solution (2.5 g) dissolved in 100 mL orthophosphoric acid, acetic acid, and water (15:60:25, v: v: v). The reaction was allowed to proceed for 1 h in a boiling water bath; the developed dye was extracted with 1 mL of toluene. After that, the absorbance was measured at a wavelength of 528 nm using a UV–vis spectrophotometer (Shimadzu UV–vis 1800). Proline concentration in the samples was determined using a standard curve established with proline solutions ranging in concentration from 0.001 to 0.005 mg mL^−1^.

### 2.8. Statistical Analysis

The data were analyzed by two-way ANOVA using R software, and Tukey’s test compared means at a 5% probability level.

## 3. Results

### 3.1. Growth Attributes

Except for the leaf area, the analysis of variance presented in Table 1 indicates that the impact of treatments is significant on all the other growth attributes (height of shoots, root length, number of leaves, fresh and dry weight). However, for all of the growth parameters examined, including the leaf area, the influence of the factor of salinity level and its interaction with treatments demonstrate statistical significance. Moreover, it is noticed that the significance is very high. The *p*-value of the two factors and their interactions was *p* < 0.001 for the height of shoots, root length, number of leaves, and dry weight.

#### 3.1.1. Height of Shoots

**Figure 1:**
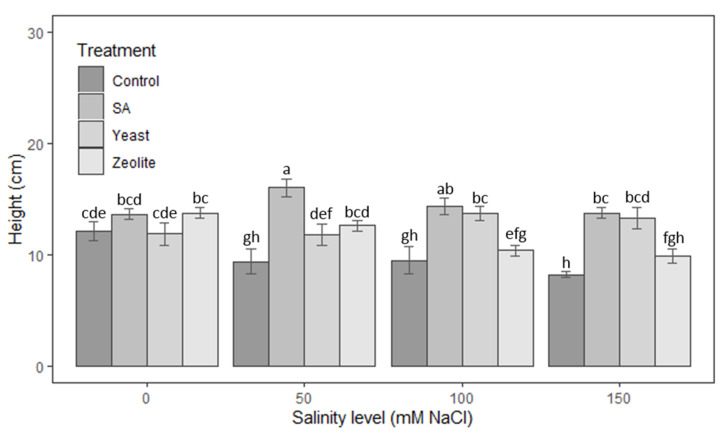
Height of shoots in lettuce plants recorded on day 50 after sowing. The plants were treated with control, salicylic acid, yeast, and zeolite at the salinity levels (0, 50, 100, 150 mM NaCl). Bars sharing the same letter among treatments are not significantly different according to Tukey’s HSD test (*p* ≤ 0.05). Errors bars represent the standard error of the mean (*n* = 4).

The results of Figure 1 indicated that SA increased plant height under salt stress compared with control, yeast, and zeolite. The zeolite treatment gives better results in the absence of salt stress, where plant heights in the zeolite-treated plot were significantly greater than in the control group (*p* < 0.05). In contrast, yeast has positively affected lettuce height at the highest salinity concentrations. At concentrations of 100 and 150 mM NaCl, the mean height values recorded in plants treated with yeast were significantly higher (*p* < 0.0001) with 45% and 61%, respectively, more than the controls. 

#### 3.1.2. Roots

**Figure 2:**
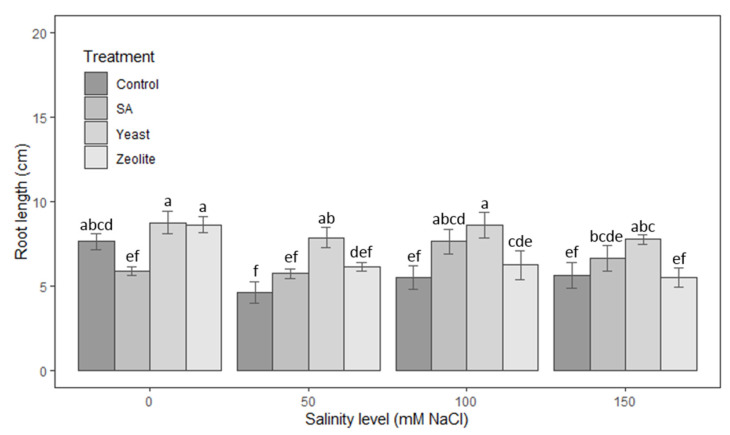
Root length in lettuce plants recorded on day 50 after sowing. The plants were treated with control, salicylic acid, yeast, and zeolite at the salinity levels (0, 50, 100, 150 Mm NaCl). Bars sharing the same letter among treatments are not significantly different according to Tukey’s HSD test (*p* ≤ 0.05). Errors bars represent the standard error of the mean (n = 4).

As shown in Figure 2, the yeast-treated lettuce has a higher salt tolerance regarding root length). Tukey’s test confirmed that the lengths in the yeast-treated plots with 100 and 150 mM NaCl were longer with 57% and 38%, respectively, than those in control (*p* < 0.005). Zeolite has a positive effect on root length in the absence of salinity treatment, but as salinity concentration rises, it loses its ability to stimulate root growth. Moreover, at the highest salt concentrations, zeolite may have an adverse effect. At the concentration of 150 mM NaCl, the mean root lengths of zeolite-treated lettuce were slightly shorter by 2% than those of the control. According to the findings, salicylic acid treatment did not stimulate the root length when there was no salt stress. The average length of roots was 23% shorter than the control (Figure 2).

#### 3.1.3. Number of Leaves 

**Figure 3:**
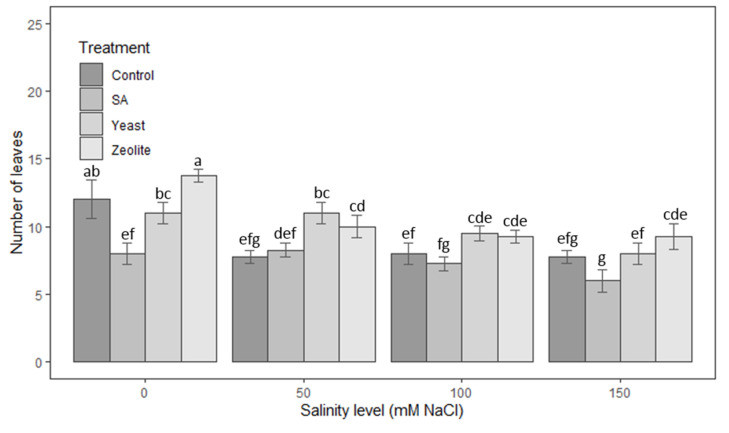
Number of leaves in lettuce plants recorded on day 50 after sowing. The plants were treated with control, salicylic acid, yeast, and zeolite at the salinity levels (0, 50, 100, 150 mM NaCl). Bars sharing the same letter among treatments are not significantly different according to Tukey’s HSD test (*p* ≤ 0.05). Errors bars represent the standard error of the mean (*n* = 4).

Zeolite increases the number of lettuce leaves in the range of 15% to 29% in comparison to untreated plots at all of the concentrations of salinity tested (0, 50, 100, 150 Mm NaCl) (Figure 3). This increase was statistically significant according to the LSD Tukey test where *p*-values were less than 0.05. Likewise, the effect of yeast was similar to that of zeolite, except that in the absence of salt stress, the number of leaves was lower than that of the control. Regarding the plants treated with salicylic acid, there was no significant difference in the number of leaves compared to the control (*p* > 0.05) in the presence of salinity at concentrations of 50 and 100 mM NaCl. Moreover, the mean number of leaves was significantly lower than the control with 33% in the absence of salt stress.

#### 3.1.4. Leaf Area

**Figure 4:**
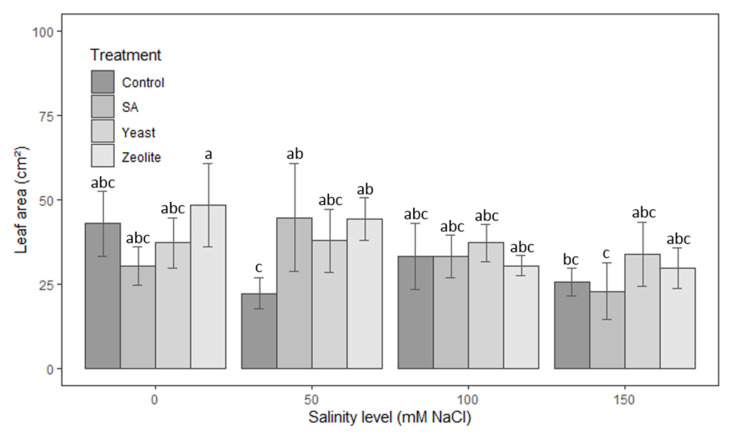
Leaf area in lettuce plants recorded on day 50 after. The plants were treated with control, salicylic acid, yeast, and zeolite at the salinity levels (0, 50, 100, 150 mM NaCl). Bars sharing the same letter among treatments are not significantly different according to Tukey’s HSD test (*p* ≤ 0.05). Error bars represent the standard error of the mean (*n* = 4).

The influence of salinity on the leaf area of lettuce in control plots cannot be easily retrieved depending on the demonstrated results (Figure 4). However, we do not notice a correlation between the concentration of salinity exerted and the leaf surface of the lettuce plants. The same observation is also valid for salicylic acid, where there is no clear link between this treatment and the foliar surface of lettuce. Although there is no statistically significant difference between all the treatments and controls at the highest salinity concentrations (100 and 150 mM NaCl), the area of leaves of lettuce plants in the plots treated with yeast was increased by 12% and 31%, respectively. At the lowest salinity concentrations 0 and 50 mM NaCl, a slight increase was observed in plots treated with zeolite.

#### 3.1.5. Fresh Weight 

**Figure 5:**
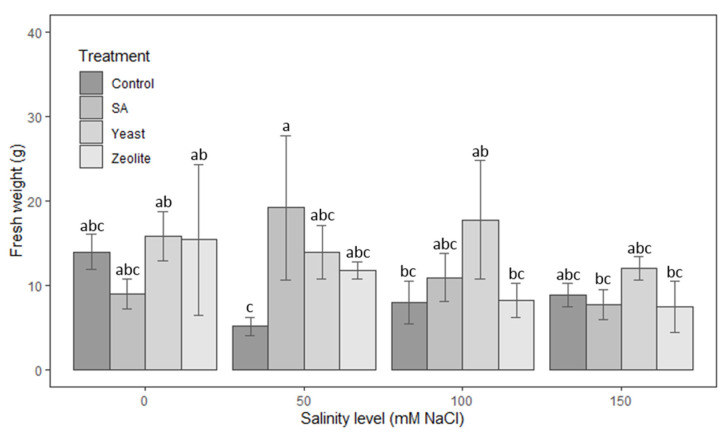
Fresh weight in lettuce plants recorded on day 50 after sowing. The plants were treated with control, salicylic acid, yeast and zeolite at the salinity levels (0, 50, 100, 150 mM NaCl). Bars sharing the same letter among treatments are not significantly different according to Tukey’s HSD test (*p* ≤ 0.05). Error bars represent the standard error of the mean (*n* = 4).

In Figure 5, it appears that zeolite treatment is positively influencing the fresh weight of lettuce at low salinity concentrations of 0 and 50 mM NaCl. However, at high salinity concentrations (100 and 150 mM NaCl), the yeast gives the best results because the mean of fresh weight was significantly more than the other treatments control, SA, and ZEO by 36%, 56%, and 62%, respectively.

#### 3.1.6. Dry Weight

**Figure 6:**
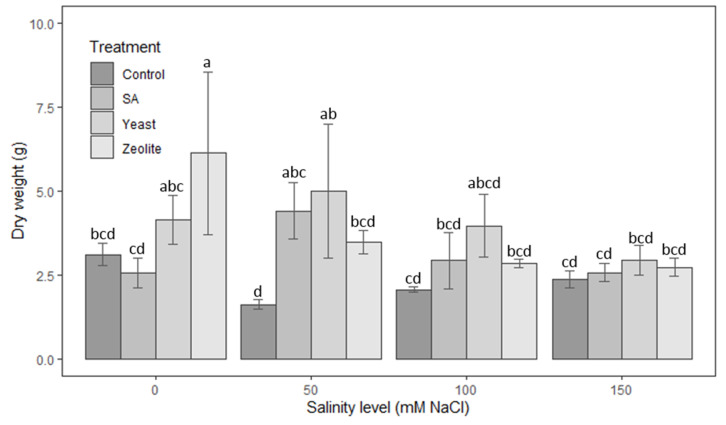
Dry weight in lettuce plants recorded on day 50 after sowing. The plants were treated with control, salicylic acid, yeast, and zeolite at the salinity levels (0, 50, 100, 150 mM NaCl). Bars sharing the same letter among treatments are not significantly different according to Tukey’s HSD test (*p* ≤ 0.05). Errors bars represent the standard error of the mean (*n* = 4).

Figure 6 shows that all treatments increase dry weight in the presence of saline stress. The yeast shows the best results where the dry weight was increased significantly (*p* < 0.05) by 33%, 207%, 92%, and 24%, respectively, at the different salinity levels of 0, 50, 100, and 150 mM NaCl compared to the control plots. Though the results of the zeolite and salicylic acid treatments do not differ significantly from that of the control at 100 and 150 mM NaCl concentrations, there was a slight increase in dry weight compared to the control. 

### 3.2. Biochemical Parameters 

As shown in Table 2, the analysis of variance reveals that all of the biochemical parameters measured including proline and sugar contents, Chl a, Chl b, and total chlorophyll were dramatically and significantly affected by the factors: treatments, salinity, and their interactions (*p* < 0.001).

#### 3.2.1. Sugar Content

When salt stress was applied, two distinct effects on sugar content were observed. In both the control and zeolite treatments, there was a positive correlation between the accumulation of sugar in the leaves and the salinity level (Table 3). While for salicylic acid and yeast treatments, a negative correlation was found. 

In the plots stressed with 150 mM NaCl, the sugar concentrations were 73%, 75%, and 23% less than the control in the salicylic acid, yeast, and zeolite treatments, respectively. Therefore, under high salt stress, none of the tested treatments increased the sugar content of lettuce leaves in comparison to the control.

#### 3.2.2. Proline Content

A positive correlation is observed between salinity level and proline concentrations in control and zeolite treatments (Table 3). The correlation is negative in the case of salicylic acid and yeast treatments. However, proline concentrations in salicylic acid-treated plots were significantly higher than all other treatments at all salinity levels tested (*p* < 0.05). The values of proline in salicylic acid treatment were 59.2% and 37.3% at 0 and 150mM NaCl, respectively, compared to the control where the values were 25.41 and 32.43 at 0 and 150 mM NaCl (Table 3.).

#### 3.2.3. Chlorophyll Content

The values of chlorophyll a and b and the total chlorophyll are presented in Table 4. 

Due to the application of salt stress (from 0 to 150 mM NaCl), the chlorophyll concentrations a and b and the total chlorophyll were reduced in control plots by 6%, 23%, and 12%, respectively. The salt stress has adversely affected the total chlorophyll content of yeast-treated plots, reducing its concentration by 30%. The concentration of chlorophyll b in yeast treatments has also remarkably decreased by 82%. However, in salicylic acid and zeolite treatments, the total chlorophyll has slightly increased by more than 5% due to salt stress. Accordingly, the total chlorophyll was positively affected by the actions of both zeolite and salicylic acid at the high salinity level (150 mM NaCl), where the concentrations of the total chlorophyll were 15% more than the control (Table 4).

## 4. Discussion

Common symptoms of damage by salt stress are growth inhibition, accelerated development and senescence, and death by long-term exposure [40]. Many studies have shown the potential to mitigate these adverse effects through using exogenous chemical or biological applications. We discuss the potential (salicylic acid, yeast, and zeolite) in tolerating salt stress on lettuce plants under field conditions.

As expected, salt stress negatively affected the root length, the height of plants, the number, and the surface of lettuce leaves. Salinity affects almost all aspects of plant development, including germination, vegetative growth, and reproductive development [41]. A remarkable reduction in plant height, tiller number, and leaf area index is also reported in rice (*Oryza sativa* L.) plants grown in saline soil [42]. A popular explanation is that salt affects plant growth and development through water stress, cytotoxicity due to an overdose of sodium (Na^+^) and chloride (Cl^−^), and nutritional imbalances [43,44]. Salt is usually associated with oxidative stress due to reactive oxygen species (ROS) [1,45,46].

The results of our experiments indicate the decrease of chlorophylls a and b and the total chlorophyll under saline conditions. Similar results were found in little hogweed (*Portulaca oleracea* L.) [47]. According to Santos [48], the reduction in chlorophyll content is attributed to the inhibition of the synthesis of 5-aminolaevulinic acid, which is a chlorophyll precursor. Therefore, it is essential to highlight that salinity reduces a leaf area, chlorophyll content, and stomatal conductance [49]. In contrast, the amounts of chlorophyll b and a were significantly higher at the highest NaCl concentration in the henna tree (*Lawsonia inermis* L.) [50].

The proline and sugar content in lettuce leaves has remarkably increased due to the salt stress in control plots. This result ties nicely with previous studies wherein salinity stress significantly increased leaf proline and soluble sugar content [47,51]. Plants accumulate organic osmolytes such as proline, betaine, polyols, sugar alcohols, and soluble sugars to tolerate osmotic stress by osmotic adjustment, which helps in turgor maintenance; detoxification of reactive oxygen species; and stabilization of the quaternary structure of proteins [52,53].

The results show that the foliar application of salicylic acid is effective in reducing salinity stress in low and mild salinity levels by promoting primary growth and chlorophyll content. In terms of root length and plant high, our results are in agreement with studies that claimed that SA could mitigate the salt stress effect on plants [54], like on cowpea (*Vigna ungiculata* L.) [17]. SA may increase ATPase activity, assisting in the reduction of sugar into a direct nutrient source that causes plants to grow taller than the control [55]. Exogenous application of salicylic acid also shows various effects on plant growth, including seed germination, budding, flowering, fruit set, and ripening in finger millet plants (*Eleusine coracana* L.) [56] and increased the fresh and dry weights in both root and shoots of wheat plants under salt stress [57]. The cause of these outcomes is that SA has a significant regulatory role in several physiological processes of plants. According to Jagendorf and Takabe [58], it can cause plants to accumulate glycinebetaine which is useful for osmoregulation in conditions of salt stress. Moreover, it is demonstrated that the application of SA on stressed plants reduces the damaging effects on cell membranes while increasing the abscisic acid content [59].

Additionally, SA may improve the resistance of plants towards abiotic stress at the genetic level by inducing several genes encoding chaperones, antioxidants, heat shock proteins (HSPs), and secondary metabolites [60].

Our findings demonstrated that SA had increased the chlorophyll and proline contents in lettuce plants. These results are consistent with that found by Khoshbakht et al. [61], who found the same trend in citrus seedlings. However, the positive effect of SA is not always obvious with respect to abiotic stress in plants [62]. Few studies have reported proline reduction by SA application in plants such as Safflower (*Carthamus tinctorius* L.) [63], chamomile (*Matricaria chamomilla*) [64], and common bean plants (*Phaseolus vulgaris* L.) [65].

For the number of leaves and sugar content, an adverse effect has been found in lettuce plants stressed by 150 mM NaCl. In contrast, Khalifa et al. [66] have reported an increase in total soluble sugars content in the same plant irrigated with saline water (3.22 dS m^−1^). In general, low or very high SA concentrations increase the susceptibility of plants to abiotic stresses. The optimal range was estimated from 0.1 to 0.5 mM, for better resistance to abiotic stress [67]. Therefore, the harmful effects of salicylic acid due to the exogenous application might have occurred in our study, which could negatively affect the sugar content and the number of leaves.

Yeast showed good tolerance to salinity stress in all growth parameters compared to control. The application of yeast improved the vegetative growth of snap beans (*Phaseolus vulgaris* L.) [68]. Similarly, yeast treatments exhibited the best plant growth and yield of pea (*Pisum sativum* L.) cv [69]. In another study, the yeasts led to the highest yield of garlic plants among five biostimulants tested [70]. The improvement in plant growth in response to foliar application of active dry yeast can be attributed to its different nutrient content higher protein and vitamin values which may play a critical role in improving growth and controlling the incidence of fungal diseases [23,26,28]. In addition, several plant growth regulators such as gibberellins and cytokines could be produced by yeast which stimulates plant physiological processes, increases cell division and expansion, and thereby increases the vegetative growth of plants [71,72,73].

Surprisingly, when it comes to the proline, sugar, and chlorophylls a and b contents, their concentrations were lower than the control which is not in agreement with the study of Darwesh [27], who claimed that regardless of the salinity level, applying yeast or amino acids on date palm trees (*Phoenix dactylifera* L.) has consistently raised the contents of chlorophyll a and b and amino acids and total sugars. Despite the low concentrations of osmoprotectants such as proline and sugar, the lettuce plants treated with yeast at a high salinity level showed a promising vegetative growth compared to the control. In some cases of rice plants grown under saline conditions, the high level of proline was considered a sign of salt injury instead of salt tolerance [74]. Similarly, the sorghum genotype sensitive to salt was found to have less proline in comparison to a tolerant genotype [75]. Therefore, a mechanism other than ion regulation may be involved in the salt tolerance of yeast-treated plants. Spraying yeast on plant leaves might provide several nutrients, vitamins, amino acids [23], and plant growth regulators [71] ready to absorb through leaves. Accordingly, foliar nutrition led to a healthy metabolic state in stressed plants which results in vigorous plant growth [76]. So, if the high concentrations of Na^+^ and Cl^−^ in the soil impaired the nutrient absorption by the roots, foliar nutrition might have offered it. 

Zeolite increased the number of leaves of lettuce significantly at all salinity levels. Still, unlike similar research, it showed a marginal effect on all other studied growth and biochemical parameters. Under salt stress conditions, zeolite can be an amendment to reduce the salinity effect [12,14,15]. The application of chitosan or zeolite counteracted the depressing effects of salinity on plant growth, photosynthetic pigments, oil percentage, and minerals of Rosemary (*Rosmarinus officinalis* L.) [77]. The foliar application of zeolite positively affected Egyptian cotton’s chemical composition, growth, boll setting, and productivity under salinity stress [78]. It is worth pointing out that most studies have tested zeolite by soil application. These studies have demonstrated the capacity of zeolite to improve soil fertility by i) zeolite absorption of Na^+^ and Cl^−^ which enter into the cavities and consequently improve the soil properties [79]; ii) improving holding water and nutrients [80], which provides an adequate supply of nutrients to the crops [81]; and iii) ensuring the buffer capacity of soils [12]. Therefore, we probably found these limited results due to using zeolite as a spray application instead of a soil application.

## 5. Conclusions

In summary, zeolite has improved the morphological characteristics of lettuce plants only in the absence or in low salinity concentrations. Accordingly, zeolite is ineffective in reducing the negative effect of salt stress. Regarding the foliar spray of salicylic acid, it has been demonstrated to be useful in mitigating salinity stress in situations of mild salinity by promoting primary growth and chlorophyll content. However, at high salinity levels, only yeast extract better enhanced the growth of lettuce plants, especially the weight and the leaf area. It is likely that this positive effect was due to several nutrients offered by yeast through leaf absorption. Future research will be essential to investigate a combination of salicylic acid and yeast application on stressed plants. It is also important to understand the mechanisms involved in the tolerance of salt stress when the yeast extract is applied.

## Figures and Tables

**Table 1:** Analysis of variance (mean square) of growth attributes.

Source	Df	Height of Shoots	Root Length	Number of Leaves	Leaf Area	Fresh Weight	Dry Weight
		(cm)	(cm)	unit	(cm^2^)	(g)	(g)
Treatment	3	59.87 ***	16.839 ***	30.64 ***	175.2 ns	97.88 **	9.582 ***
Salinity	3	7.21 ***	8.339 ***	34.89 ***	418.4 **	60.73 *	5.953 ***
Treatment × Salinity	9	8.64 ***	3.217 ***	4.47 ***	207.3 **	58.59 **	3.860 ***

Not significant (ns), or significant at *p* < 0.05 (*), *p* < 0.01 (**), *p* < 0.001 (***), analysis of variance.

**Table 2:** Analysis of variance (mean square) of biochemical parameters.

	Df	Proline Content	Sugar Content	Chlorophyll a	Chlorophyll b	Total Chlorophyll
		(µg g^−1^ FW)	(mg 100 mg^−1^ FW)	(mg g^−1^ FW)	(mg g^−1^ FW)	(mg g^−1^ FW)
Treatment	3	1786.0 ***	0.03126 ***	0.004967 ***	0.011809 ***	0.0019023 ***
Salinity	3	100.7 ***	0.00271 ***	0.000149 ***	0.002272 ***	0.0028026 ***
Treatment × Salinity	9	191.5 ***	0.04481 ***	0.000391 ***	0.001427 ***	0.0025205 ***

Not significant (ns), or significant at *p* < 0.001 (***), analysis of variance.

**Table 3:** Effect of control, salicylic acid, yeast, and zeolite treatments on sugar and proline contents (µg 100 mg^−1^ mg of fresh weight) at different salinity levels (0, 50, 100, 150 mM NaCl). The measurements were performed 50 days after sowing.

Treatment	Salinity Level (mM NaCl)	Content, as Mean ± S.E.	
		Proline (µg g^−1^ FW)	Sugar (mg 100 mg^−1^ FW)
Control	0	25,500 ± 0.064 g	0.521 ± 0.011 a
	50	26,200 ± 0.065 g	0.366 ± 0.011 bcd
	100	28,800 ± 0.897 f	0.363 ± 0.014 cd
	150	31,900 ± 1.150 e	0.235 ± 0.011 fg
Salicylic acid	0	60,300 ± 1.510 a	0.346 ± 0.011 de
	50	54,700 ± 1.260 b	0.318 ± 0.016 e
	100	38,200 ± 0.293 c	0.272 ± 0.008 f
	150	37,500 ± 0.247 c	0.138 ± 0.015 i
Yeast	0	34,400 ± 0.905 d	0.380 ± 0.012 bcd
	50	25,100 ± 0.195 g	0.363 ± 0.007 cd
	100	22,500 ± 0.225 h	0.185 ± 0.015 h
	150	15,800 ± 0.497 j	0.126 ± 0.016 i
Zeolite	0	13,800 ± 1.110 j	0.403 ± 0.017 b
	50	19,800 ± 0.462 i	0.393 ± 0.009 bc
	100	20,900 ± 0.816 hi	0.272 ± 0.006 f
	150	25,700 ± 0.362 g	0.231 ± 0.017 g

Different letters between means in the same columns denote significant differences (Tukey test, *p* < 0.05).

**Table 4:** The concentration of chlorophyll a, chlorophyll b, and total chlorophyll in lettuce plants treated with control, salicylic acid, yeast, and zeolite at the salinity levels (0, 50, 100, 150 mM NaCl). The measurements were performed 50 days after sowing.

Treatment	Salinity Level (mM NaCl)	Content, as Mean ± S.E. (in mg g^−^^1^ FW)		
		Chlorophyll a	Chlorophyll b	Total Chlorophyll
Control	0	0.229 ± 0.003 cd	0.136 ± 0.004 a	0.364 ± 0.007 bc
	50	0.224 ± 0.004 cde	0.125 ± 0.003 bc	0.349 ± 0.007 cd
	100	0.223 ± 0.004 cde	0.116 ± 0.002 cd	0.339 ± 0.006 d
	150	0.215 ± 0.005 e	0.104 ± 0.004 e	0.320 ± 0.008 e
Salicylic acid	0	0.228 ± 0.004 cd	0.122 ± 0.004 bcd	0.368 ± 0.005 b
	50	0.232 ± 0.002 c	0.121 ± 0.003 bcd	0.356 ± 0.005 bcd
	100	0.232 ± 0.003 c	0.125 ± 0.003 bc	0.353 ± 0.005 bcd
	150	0.229 ± 0.002 cd	0.138 ± 0.003 a	0.350 ± 0.007 cd
Yeast	0	0.283 ± 0.004 a	0.123 ± 0.002 bc	0.406 ± 0.005 a
	50	0.264 ± 0.004 b	0.053 ± 0.002 f	0.317 ± 0.006 e
	100	0.262 ± 0.002 b	0.043 ± 0.003 g	0.305 ± 0.004 e
	150	0.262 ± 0.003 b	0.022 ± 0.002 h	0.285 ± 0.004 f
Zeolite	0	0.224 ± 0.003 cde	0.126 ± 0.002 b	0.370 ± 0.007 b
	50	0.220 ± 0.001 de	0.124 ± 0.003 bc	0.350 ± 0.004 cd
	100	0.222 ± 0.003 de	0.121 ± 0.002 bcd	0.344 ± 0.004 d
	150	0.256 ± 0.003 b	0.114 ± 0.003 d	0.343 ± 0.006 d

Different letters between means in the same columns denote significant differences (Tukey test, *p* < 0.05).

## Data Availability

The original contributions presented in the study are included in the article further inquiries can be directed to the corresponding author.

## References

[1] Youssef M.H.M., Raafat A., El-Yazied A.A., Selim S., Azab E., Khojah E., El Nahhas N., Ibrahim M.F.M. (2021). Exogenous Application of Alpha-Lipoic Acid Mitigates Salt-Induced Oxidative Damage in Sorghum Plants through Regulation Growth, Leaf Pigments, Ionic Homeostasis, Antioxidant Enzymes, and Expression of Salt Stress Responsive Genes. Plants.

[2] Dajic Z. (2006). Salt stress. Physiology and Molecular Biology of Stress Tolerance in Plants.

[3] Munns R., Tester M. (2008). Mechanisms of salinity tolerance. Annu. Rev. Plant Biol..

[4] Mao W., Zhu Y., Wu J., Ye M., Yang J. (2022). Evaluation of effects of limited irrigation on regional-scale water movement and salt accumulation in arid agricultural areas. Agric. Water Manag..

[5] Pitman M.G., Läuchli A., Läuchli A., Lüttge U. (2004). Global impact of salinity and agricultural ecosystems. Salinity: Environment—Plants—Molecules.

[6] Alsamadany H., Mansour H., Elkelish A., Ibrahim M.F. (2022). Folic Acid Confers Tolerance against Salt Stress-Induced Oxidative Damages in Snap Beans through Regulation Growth, Metabolites, Antioxidant Machinery and Gene Expression. Plants.

[7] El Nahhas N., AlKahtani M.D., Abdelaal K.A., Al Husnain L., AlGwaiz H.I., Hafez Y.M., Attia K.A., El-Esawi M.A., Ibrahim M.F., Elkelish A. (2021). Biochar and jasmonic acid application attenuates antioxidative systems and improves growth, physiology, nutrient uptake and productivity of faba bean (*Vicia faba* L.) irrigated with saline water. Plant Physiol. Biochem..

[8] Ramadan K.M.A., Alharbi M.M., Alenzi A.M., El-Beltagi H.S., Darwish D.B., Aldaej M.I., Shalaby T.A., Mansour A.T., El-Gabry Y.A., Ibrahim M.F.M. (2022). Alpha Lipoic Acid as a Protective Mediator for Regulating the Defensive Responses of Wheat Plants against Sodic Alkaline Stress: Physiological, Biochemical and Molecular Aspects. Plants.

[9] Negrão S., Schmöckel S.M., Tester M. (2017). Evaluating physiological responses of plants to salinity stress. Ann. Bot..

[10] Costa S.F., Martins D., Agacka-Mołdoch M., Czubacka A., de Sousa Araújo S., Kumar V., Wani S.H., Suprasanna P., Tran L.-S.P. (2018). Strategies to alleviate salinity stress in plants. Salinity Responses and Tolerance in Plants, Volume 1.

[11] Zheng J., Chen T., Wu Q., Yu J., Chen W., Chen Y., Siddique K.H.M., Meng W., Chi D., Xia G. (2018). Effect of zeolite application on phenology, grain yield and grain quality in rice under water stress. Agric. Water Manag..

[12] Yuvaraj M., Subramanian K.S. (2016). Zeolites application in agriculture. Adv. Life Sci..

[13] Ntanos E., Kekelis P., Assimakopoulou A., Gasparatos D., Denaxa N.-K., Tsafouros A., Roussos P.A. (2021). Amelioration effects against salinity stress in strawberry by bentonite–zeolite mixture, glycine betaine, and *Bacillus amyloliquefaciens* in terms of plant growth, nutrient content, soil properties, yield, and fruit quality characteristics. Appl. Sci..

[14] Negahban M., Saeedfar S., Ramezan D., Asil M.H. (2014). Effects of natural zeolite to reduce salt stress in kentucky bluegrass (*Poa pratensis*). Russ. J. Biol. Res..

[15] Aboul-Magd M., Elzopy K.A., Zangana Z.R.M. (2020). Effect of zeolite and urea fertilizer on maize grown under saline conditions. Middle East J. Appl. Sci..

[16] Koo Y.M., Heo A.Y., Choi H.W. (2020). Salicylic acid as a safe plant protector and growth regulator. Plant Pathol. J..

[17] El-Taher A.M., Abd El-Raouf H.S., Osman N.A., Azoz S.N., Omar M.A., Elkelish A., Abd El-Hady M.A.M. (2022). Effect of Salt Stress and Foliar Application of Salicylic Acid on Morphological, Biochemical, Anatomical, and Productivity Characteristics of Cowpea (*Vigna unguiculata* L.) Plants. Plants.

[18] Ghafoor M., Ali Q., Malik A. (2020). Effects of salicylic acid priming for salt stress tolerance in wheat. Biol. Clin. Sci. Res. J..

[19] Khodary S.E.A. (2004). Effect of salicylic acid on the growth, photosynthesis and carbohydrate metabolism in salt stressed maize plants. Int. J. Agric. Biol..

[20] Patel P.K., Hemantaranjan A. (2012). Salicylic acid induced alteration in dry matter partitioning, antioxidant defence system and yield in chickpea (*Cicer arietinum* L.) under drought stress. Asian J. Crop Sci..

[21] Abd-Alrahman H.A., Aboud F.S. (2021). Response of sweet pepper plants to foliar application of compost tea and dry yeast under soilless conditions. Bull. Natl. Res. Cent..

[22] Dawood M.G., Sadak M.S., Abdallah M.M.S., Bakry B.A., Darwish O.M. (2019). Influence of biofertilizers on growth and some biochemical aspects of flax cultivars grown under sandy soil conditions. Bull. Natl. Res. Cent..

[23] Lonhienne T., Mason M.G., Ragan M.A., Hugenholtz P., Schmidt S., Paungfoo-Lonhienne C. (2014). Yeast as a biofertilizer alters plant growth and morphology. Crop Sci..

[24] Kang S.-M., Radhakrishnan R., You Y.-H., Khan A.L., Park J.-M., Lee S.-M., Lee I.-J. (2015). Cucumber performance is improved by inoculation with plant growth-promoting microorganisms. Acta Agric. Scand. Sect. B—Soil Plant Sci..

[25] Marzauk N.M. (2014). Effect of vitamin E and Yeast extract foliar application on growth, pod yield and both green pod and seed yield of broad bean. Middle East J. Appl. Sci..

[26] Francesca S., Arena C., Hay Mele B., Schettini C., Ambrosino P., Barone A., Rigano M.M. (2020). The use of a plant-based biostimulant improves plant performances and fruit quality in tomato plants grown at elevated temperatures. Agronomy.

[27] Darwesh R.S.S. (2013). Improving growth of date palm plantlets grown under salt stress with yeast and amino acids applications. Ann. Agric. Sci..

[28] El-Yazied A.A., Mady M.A. (2012). Effect of boron and yeast extract foliar application on growth, pod setting and both green pod and seed yield of broad bean (*Vicia faba* L.). J. Am. Sci..

[29] Rehman A., Hassan F., Qamar R., Rehman A.U. (2021). Application of plant growth promoters on sugarcane (*Saccharum officinarum* L.) budchip under subtropical conditions. Asian J. Agric. Biol..

[30] Enebe M.C., Babalola O.O. (2018). The influence of plant growth-promoting rhizobacteria in plant tolerance to abiotic stress: A survival strategy. Appl. Microbiol. Biotechnol..

[31] Aćamović-DJoković G., Pavlović R., Mladenović J., DJurić M. (2011). Vitamin C content of different types of lettuce varieties. Acta Agric. Serbica.

[32] Kim M.J., Moon Y., Tou J.C., Mou B., Waterland N.L. (2016). Nutritional value, bioactive compounds and health benefits of lettuce (*Lactuca sativa* L.). J. Food Compos. Anal..

[33] Medina-Lozano I., Bertolín J.R., Díaz A. (2021). Nutritional value of commercial and traditional lettuce (*Lactuca sativa* L.) and wild relatives: Vitamin C and anthocyanin content. Food Chem..

[34] Mou B. (2012). Nutritional quality of lettuce. Curr. Nutr. Food Sci..

[35] Noumedem J.A.K., Djeussi D.E., Hritcu L. (2017). *Lactuca sativa*. Medicinal Spices and Vegetables from Africa.

[36] Maroyi A. (2014). Not just minor wild edible forest products: Consumption of pteridophytes in sub-Saharan Africa. J. Ethnobiol. Ethnomed..

[37] Arnon D.I. (1949). Copper enzymes in isolated chloroplasts. Polyphenoloxidase in *Beta vulgaris*. Plant Physiol..

[38] Dubois M., Gilles K.A., Hamilton J.K., Rebers P.T., Smith F. (1956). Colorimetric method for determination of sugars and related substances. Anal. Chem..

[39] Troll W., Lindsley J. (1955). A photometric method for the determination of proline. J. Biol. Chem..

[40] Zhu J. (2007). Plant salt stress. Encyclopedia of Life Sciences (ELS).

[41] Chinnusamy V., Zhu J., Zhu J.-K., Setlow J.K. (2006). Salt stress signaling and mechanisms of plant salt tolerance. Genetic Engineering.

[42] Hasamuzzaman M., Fujita M., Islam M.N., Ahamed K.U., Nahar K. (2009). Performance of four irrigated rice varieties under different levels of salinity stress. Int. J. Integr. Biol..

[43] Farooq M., Hussain M., Wakeel A., Siddique K.H.M. (2015). Salt Stress in Maize: Effects, resistance mechanisms, and management. A Review. Agron. Sustain. Dev..

[44] Munns R., James R.A., Läuchli A. (2006). Approaches to increasing the salt tolerance of wheat and other cereals. J. Exp. Bot..

[45] Oukarroum A., Bussotti F., Goltsev V., Kalaji H.M. (2015). Correlation between reactive oxygen species production and photochemistry of photosystems I and II in *Lemna gibba* L. Plants under salt stress. Environ. Exp. Bot..

[46] Schmidt R., Mieulet D., Hubberten H.-M., Obata T., Hoefgen R., Fernie A.R., Fisahn J., San Segundo B., Guiderdoni E., Schippers J.H. (2013). Salt-responsive ERF1 regulates reactive oxygen species–dependent signaling during the initial response to salt stress in rice. Plant Cell.

[47] Hnilickova H., Kraus K., Vachova P., Hnilicka F. (2021). Salinity stress affects photosynthesis, malondialdehyde formation, and proline content in *Portulaca oleracea* L. Plants.

[48] Santos C.V. (2004). Regulation of chlorophyll biosynthesis and degradation by salt stress in sunflower leaves. Sci. Hortic..

[49] Netondo G.W., Onyango J.C., Beck E. (2004). Sorghum and Salinity: II. Gas exchange and chlorophyll fluorescence of sorghum under salt stress. Crop Sci..

[50] Najar B., Pistelli L., Marchioni I., Pistelli L., Muscatello B., De Leo M., Scartazza A. (2020). Salinity-Induced Changes of Photosynthetic Performance, Lawsone, VOCs, and antioxidant metabolism in *Lawsonia Inermis* L. Plants.

[51] Ahmed S., Ahmed S., Roy S.K., Woo S.H., Sonawane K.D., Shohael A.M. (2019). Effect of salinity on the morphological, physiological and biochemical properties of lettuce (*Lactuca sativa* L.) in Bangladesh. Open Agric..

[52] Bohnert H.J., Jensen R.G. (1996). Strategies for engineering water-stress tolerance in plants. Trends Biotechnol..

[53] Munir N., Hasnain M., Roessner U., Abideen Z. (2021). Strategies in improving plant salinity resistance and use of salinity resistant plants for economic sustainability. Crit. Rev. Environ. Sci. Technol..

[54] Yildirim E., Turan M., Guvenc I. (2008). Effect of foliar salicylic acid applications on growth, chlorophyll, and mineral content of cucumber grown under salt stress. J. Plant Nutr..

[55] Iqbal N., Nazar R., Khan M.I.R., Masood A., Kahan N.A. (2011). Role of gibberellins in regulation of source-sink relations under optimal and limiting environmental conditions. Curr. Sci..

[56] Appu M., Muthukrishnan S. (2014). Foliar application of salicylic acid stimulates flowering and induce defense related proteins in finger millet plants. Univers. J. Plant Sci..

[57] Erdal S., Aydın M., Genisel M., Taspınar M.S., Dumlupinar R., Kaya O., Gorcek Z. (2011). Effects of salicylic acid on wheat salt sensitivity. Afr. J. Biotechnol..

[58] Jagendorf A.T., Takabe T. (2001). Inducers of glycinebetaine synthesis in barley. Plant Physiol..

[59] Bandurska H., Stroinski A. (2005). The effect of salicylic acid on barley response to water deficit. Acta Physiol. Plant..

[60] Jumali S.S., Said I.M., Ismail I., Zainal Z. (2011). Genes induced by high concentration of salicylic acid in ‘*Mitragyna speciosa*’. Aust. J. Crop Sci..

[61] Khoshbakht D., Asgharei M.R. (2015). Influence of foliar-applied salicylic acid on growth, gas-exchange characteristics, and chlorophyll fluorescence in citrus under saline conditions. Photosynthetica.

[62] Horváth E., Csiszár J., Gallé Á., Poór P., Szepesi Á., Tari I. (2015). Hardening with salicylic acid induces concentration-dependent changes in abscisic acid biosynthesis of tomato under salt stress. J. Plant Physiol..

[63] Shaki F., Maboud H.E., Niknam V. (2018). Growth enhancement and salt tolerance of safflower (*Carthamus tinctorius* L.), by salicylic acid. Curr. Plant Biol..

[64] Kováčik J., Klejdus B., Hedbavny J., Bačkor M. (2009). Salicylic acid alleviates NaCl-induced changes in the metabolism of *Matricaria chamomilla* plants. Ecotoxicology.

[65] Palma F., Lluch C., Iribarne C., García-Garrido J.M., Tejera García N.A. (2009). Combined effect of salicylic acid and salinity on some antioxidant activities, oxidative stress and metabolite accumulation in *Phaseolus vulgaris*. Plant Growth Regul..

[66] Khalifa G.S., Abdelrassoul M., Hegazi A.M., Elsherif M.H. (2016). Attenuation of negative effects of saline stress in two lettuce cultivars by salicylic acid and glycine betaine. Gesunde Pflanz..

[67] Hasanuzzaman M., Nahar K., Fujita M., Azooz M.M., Prasad M.N.V. (2013). Plant response to salt stress and role of exogenous protectants to mitigate salt-induced damages. Ecophysiology and Responses of Plants under Salt Stress.

[68] El-Greadly N. (2007). physiological responses, growth, yield and quality of snap beans in response to foliar application of yeast, vitamin E and zinc under sandy soil conditions. Aust. J. Basic Appl. Sci..

[69] Zaghloul R.A., Abou-Aly H.E., El-Meihy R.M., El-Saadony M.T. (2015). Improvement of growth and yield of pea plants using integrated fertilization management. Univers. J. Agric. Res..

[70] Shalaby T.A., El-Ramady H. (2014). Effect of foliar application of bio-stimulants on growth, yield, components, and storability of garlic (*Allium sativum* L.). Aust. J. Crop Sci..

[71] Fu S.-F., Sun P.-F., Lu H.-Y., Wei J.-Y., Xiao H.-S., Fang W.-T., Cheng B.-Y., Chou J.-Y. (2016). Plant Growth-Promoting traits of yeasts isolated from the phyllosphere and rhizosphere of *Drosera spatulata* Lab. Fungal Biol..

[72] Mukherjee A., Verma J.P., Gaurav A.K., Chouhan G.K., Patel J.S., Hesham A.E.-L. (2020). Yeast a potential bio-agent: Future for plant growth and postharvest disease management for sustainable agriculture. Appl. Microbiol. Biotechnol..

[73] Taha R.S., Seleiman M.F., Alhammad B.A., Alkahtani J., Alwahibi M.S., Mahdi A.H.A. (2020). Activated yeast extract enhances growth, anatomical structure, and productivity of *Lupinus termis* L. plants under actual salinity conditions. Agronomy.

[74] Lutts S., Majerus V., Kinet J.M. (1999). NaCl effects on proline metabolism in rice (*Oryza sativa*) seedlings. Physiol. Plant..

[75] De Lacerda C.F., Cambraia J., Oliva M.A., Ruiz H.A., Prisco J.T. (2003). Solute accumulation and distribution during shoot and leaf development in two sorghum genotypes under salt stress. Environ. Exp. Bot..

[76] Batool S., Khan S., Basra S.M.A. (2020). Foliar application of moringa leaf extract improves the growth of moringa seedlings in winter. South Afr. J. Bot..

[77] Helaly M., Farouk S., Arafa S., Amhimmid N. (2018). Inducing salinity tolerance of rosemary (*Rosmarinus officinalis* L.) plants by chitosan or zeolite application. AJAAR.

[78] El-Gabiery A.E., Ata Allah Y.F.A. (2017). Effect of foliar application with bentonite on growth and productivity of egyptian cotton. J. Plant Prod..

[79] Ferretti G., Di Giuseppe D., Faccini B., Coltorti M. (2018). Mitigation of sodium risk in a sandy agricultural soil by the use of natural zeolites. Environ. Monit. Assess.

[80] Hazrati S., Tahmasebi-Sarvestani Z., Mokhtassi-Bidgoli A., Modarres-Sanavy S.A.M., Mohammadi H., Nicola S. (2017). Effects of zeolite and water stress on growth, yield and chemical compositions of *Aloe vera* L. Agric. Water Manag..

[81] Gholamhoseini M., AghaAlikhani M., Dolatabadian A., Khodaei-Joghan A., Zakikhani H. (2012). Decreasing nitrogen leaching and increasing canola forage yield in a sandy soil by application of natural zeolite. Agron. J..

